# Seasonal variation of hemato-biochemical parameters in indigenous pig: Zovawk of Mizoram

**DOI:** 10.14202/vetworld.2015.732-737

**Published:** 2015-06-17

**Authors:** P. Mayengbam, T. C. Tolenkhomba

**Affiliations:** Department of Veterinary Physiology and Biochemistry, College of Veterinary Sciences and Animal Husbandry, Central Agricultural University, Selesih, Aizawl, Mizoram, India

**Keywords:** enzyme, electrolyte, hematology, Mizoram, pig, Zovawk

## Abstract

**Aim:**

To investigate the influence of season on erythrocyte picture, plasma enzyme and electrolyte profile of local pig of Mizoram at a different age.

**Materials and Methods:**

A volume of 2 ml of blood samples were collected from 72 Zovawk pigs of three different age groups *viz*. pre-weaning, grower and adult pigs reared in College of Veterinary Sciences and Animal Husbandry Selesih, Aizawl, Mizoram, India, livestock farm during summer and winter in order to find out influence of season on erythrocyte picture, enzyme and electrolyte profile. The hematological parameters were estimated by using an automatic blood analyzer. The biochemical parameters were estimated by using diagnostic kits.

**Results:**

The grower pigs had lower hemoglobin (Hb), packed cell volume (PCV), mean corpuscular volume (MCV), mean corpuscular Hb (MCH) during summer, rainy and winter seasons (p<0.05) and lower MCH concentration (MCHC) during summer season (p<0.05). Adult pigs had lower Hb with higher total erythrocyte count in rainy season. PCV and MCV of adult pigs decreased in summer (p<0.05). Serum glutamic oxaloacetic transaminase activity of both the grower and adult groups increased during summer and rainy seasons (p<0.05). Zovawk pigs had higher sodium (Na) and lower potassium (K) in rainy season (p<0.05). Pre-weaning piglets had higher Na, K, calcium (Ca), and magnesium (Mg) in summer than in winter. Grower pigs had higher Na, K and Ca in summer (p<0.05). Pi of pre-weaning and grower groups was higher in winter. Adult pigs had lower alkaline phosphatase activity and Ca in summer and higher Pi and Mg in winter (p<0.05).

**Conclusion:**

Seasonal variation in some hemato-biochemical parameters of Zovawk pig were investigated. Influence of season on the hemato-biochemical profile was most evident during the grower stage, followed by adults and pre-weaning piglets.

## Introduction

Physiological signs of heat stress in pigs include an increase in respiration rate, rectal temperature, pulse rate, reduction in feed intake and behavioral adjustments. Heat stress is one of the major concerns in pig farming during summer as pigs do not have functional sweat glands to remove efficiently body heat, unlike other livestock species. Pigs adopt evaporative heat loss mechanism in order to dissipate heat during heat load. High humidity causes physiological signs of heat stress at lower ambient temperature, and the impact of heat stress is more when high humidity exists in the presence of high ambient temperature [1,2]. Under the heat stress, penalties to efficient performance, production, reproduction, feed conversion, health and welfare of animals can be severe as it is well-documented [3,4]. A number of hematological and biochemical parameters also showed variations in the presence of stress due to high ambient temperature and relative humidity [4,5].

The Zovawk pigs whose home tract is in different parts of Mizoram state in India occupies only 47,143 out of total pig population of 167,361 in Mizoram [6]. Scientific report on proximate component analysis of body measurements suggested that a few body measurement of Zovawk pigs could be exploited in breeding and selection programs in order to increase its population and also its productivity [7]. Recently, Zovawk pigs had been reported to have normally higher content of hemoglobin (Hb) and total erythrocyte count (TEC) [8] like other indigenous pigs [9,10] and some wild and captive pigs [11,12] which were higher than those reported in other exotic and crossbred pigs [10,13-15]. The content of plasma Na in Zovawk was found to be higher [16] as compared to that of other indigenous, exotic and crossbred pigs [13,15,17]. There is however no literature available to explain the seasonal changes in hematology or blood biochemical profile of pigs domesticated in Mizoram and more particularly the Zovawk pigs of Mizoram.

Mizoram is situated between 92015′′-93029′′ E longitude and 21058′′-24035′′ N latitude with average elevation 1132 m above the mean sea level. The state enjoys a pleasant climate throughout the year except in the summer and rainy seasons during which a very high relative humidity prevails [18]. The present study was therefore undertaken in order to investigate seasonal variation on erythrocyte picture, plasma enzyme profile and electrolyte status of indigenous pig – Zovawk. The presented data could be utilized as basic information about this kind of pig and application of knowledge in amelioration of stress to Zovawk pigs under conventional rearing system and more particularly to management practices in a state like Mizoram where relative humidity is very high during the summer, which will ultimately facilitate in averting the economic losses.

## Materials and Methods

### Ethical approval

The present investigation was carried out after the approval of the Institutional Animal Ethics Committee.

### Animals and blood sampling

The study was carried out in Zovawk pig of Mizoram reared in the Livestock Farm, College of Veterinary Sciences and Animal Husbandry, Central Agricultural University, Selesih, Aizawl, Mizoram, India. The study comprised of three different groups, pre-weaning group (1 month old), grower group (2.5 months old) and adults (12-14 months old) and each groups comprised of 12 animals of either sexes. The piglets were kept with the mother till the weaning of the piglets on 56^th^ day thereafter they were being given grower diets. The adult female pigs were in the first parity and the adult males had given 1^st^ service.

The body weight of animals was recorded 1 day before the blood sampling. The average body weight of pigs used in the present investigation in pre-weaning, grower and adults groups were 2.39±0.14 kg, 4.98±0.24 kg and 59.22±1.73 kg, respectively. Blood sampling was done by puncturing anterior venacava in heparinized vacutainers. Blood samples were collected in three different periods i.e., summer, rainy and winter seasons. Cold chain was maintained during the transit of the samples from farm to the laboratory.

### Collection of meteorological data

Three different seasons of the calendar year were selected *viz*. summer season (March-June), rainy season (July-October) and winter season (November-February). Meteorological data were recorded from the automatic weather Station (WatchDog, Spectrum Technologies, Inc.) located inside the campus of College of Veterinary Sciences and Animal Husbandry, Central Agricultural University, Selesih, Aizawl, Mizoram, India. The average minimum ambient temperature (T_min._), average maximum ambient temperature (T_max._), average minimum relative humidity (R.H_min._), average maximum relative humidity (R.H_max._) and temperature humidity index (THI) were calculated and presented in Table-1. THI was calculated by using the following formula:

**Table-1:** Ambient temperature, relative humidity and THI in summer, rainy and winter season at Selesih, Aizawl.

Season	Average ambient temperature (°C)		Average relative humidity (%)		Average THI
	
	T_min_	T_max_	R.H_min_	R.H_max_	
Summer	19.66	28.67	50.75	93.75	72.79
Rainy	20.63	28.87	71.50	97.75	74.96
Winter	14.82	25.51	49.50	84.50	66.40

THI=Temperature humidity index


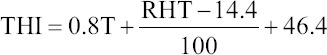


Where, T and RH are average ambient temperature and average relative humidity respectively [19].

### Analysis of samples

Hematological parameters were analyzed by using VetscanHM5 immediately after collection. Plasma was separated from whole blood by centrifugation at 3000 rpm for 20 min immediately after collection of blood. All the parameters *viz*. serum glutamic-oxaloacetic transaminase (SGOT), serum glutamic pyruvic transaminase (SGPT), alkaline phosphatase (ALP), sodium (Na), potassium (K), chloride (Cl), calcium (Ca), phosphorus (Pi) and magnesium (Mg) were analyzed in the plasma. All the parameters were estimated by using diagnostic kits from M/s Crest Biosystems, India by following standard protocols (*viz*. SGOT and SGPT by Reitman and Frankel’s method, ALP by modified Kind and King’s method, Na and K by colorimetric method, Cl by Thiocyanate method, Ca by o-cresolphthalein complexone method, Pi by Molybdate U.V. method, and Mg by Calmagite method) by using a UV-Vis Spectrophotometer (Chemito-Spectroscan 2600).

### Statistical analysis

Data were analyzed using the SYSTAT 12 by applying independent t-test to compare between the seasons and one-way ANOVA, followed by Fisher’s least significant difference test for *post-hoc* multiple comparisons to evaluate the effect of different age groups on hematological parameters.

## Results

The seasonal variation in mean±standard error of erythrocyte picture, enzyme profile and electrolyte profile of Zovawk pig in three different groups *viz*. pre-weaning, grower and adult groups are presented in Tables 2-4 respectively.

**Table-2:** Effect of season on hematological profile of Zovawk in different age groups.

Parameter	Season	Pre-weaning	Grower	Adult
TEC (×10^6^/µl)	Summer	8.09±0.48^b^	9.84±0.27^a^	8.78±0.35^ab^^B^
	Rainy	8.14±0.15^c^	10.24±0.65^b^	16.11±1.0^a^^A^
	Winter	8.10±0.17^b^	10.66±0.54^a^	8.84±0.46^b^^B^
Hb (g/dl)	Summer	13.03±3.02^b^	12.91±0.33^b^^B^	16.76±0.32^a^^A^
	Rainy	13.67±0.23^a^	12.78±0.72^ab^^B^	11.33±1.15^b^^B^
	Winter	13.53±0.22^b^	17.75±0.83^a^^A^	17.36±1.09^a^^A^
PCV (%)	Summer	44.54±2.92^b^	43.28±0.99^b^^B^	53.15±1.93^a^^B^
	Rainy	45.15±0.73^b^	41.86±2.28^b^^B^	60.10±2.87^a^^AB^
	Winter	45.31±0.62^b^	55.85±2.64^a^^A^	60.42±2.65^a^^A^
MCV (fl)	Summer	56.11±3.44^a^	44.19±1.13^b^^B^	61.07±1.97^a^^B^
	Rainy	55.58±0.45^a^	41.83±2.35^b^^B^	60.44±0.99^a^^B^
	Winter	56.08±0.63^b^	52.58±0.62^b^^A^	69.17±3.39^a^^A^
MCH (pg)	Summer	16.37±0.97^b^	13.17±0.32^c^^B^	19.48±1.04^a^^AB^
	Rainy	16.82±0.21^a^	12.67±0.57^b^^B^	17.62±0.35^a^^B^
	Winter	16.73±0.17^b^	16.69±0.26^b^^A^	20.21±1.09^a^^A^
MCHC (%)	Summer	29.29±0.54	29.82±0.20^B^	31.93±1.22
	Rainy	30.29±0.32	30.52±0.55^AB^	29.21±0.55
	Winter	29.84±0.28	31.79±0.38^A^	29.01±1.62

a-c Values in the same row with different superscripts differ significantly (p<0.05),

A-B Values in the same column with different subscripts differ significantly (p<0.05),

TEC=Total erythrocyte count, Hb=Hemoglobin, PCV=Packed cell volume, MCV=Mean corpuscular volume, MCH=Mean corpuscular hemoglobin, MCHC=Mean corpuscular hemoglobin concentration

**Table-3:** Effect of season on SGOT, SGPT and ALP profile of Zovawk in different age groups.

Parameter	Season	Pre-weaning	Grower	Adult
SGOT (U/L)	Summer	37.25±4.63_b_	47.63±1.47_a__A_	54.50±2.24_a__A_
	Rainy	36.29±4.16_b_	52.92±2.66_a__A_	59.33±3.00_a__A_
	Winter	38.88±4.76	35.90±3.02_B_	37.58±3.81_b_
SGPT (U/L)	Summer	23.33±2.01_b_	28.13±1.46_b__A_	42.08±2.86_a_
	Rainy	22.83±2.33_a_	23.92±0.76_a__B_	40.50±3.10_b_
	Winter	22.21±2.58_b_	25.62±1.58_b__AB_	47.83±2.49_a_
ALP (Ka)	Summer	36.03±3.49_a_	15.09±1.45_b__AB_	4.93±0.20_c__B_
	Rainy	29.69±3.19_a_	20.11±2.83_b__A_	8.74±0.87_c__A_
	Winter	36.85±2.94_a_	14.29±0.98_b__B_	8.79±1.11_c__A_

a-c Values in the same row with different superscripts differ significantly (p<0.05),

A-B Values in the same column with different subscripts differ significantly (p<0.05),

SGOT=Serum glutamic oxaloacetic transaminase, SGPT=Serum glutamic pyruvic transaminase, ALP=Alkaline phosphatase

**Table-4:** Effect of season on electrolyte profile of Zovawk in different age groups.

Parameter	Season	Pre-weaning	Grower	Adult
Sodium (mmol/L)	Summer	166.04±5.03_B_	169.77±3.81_B_	172.59±5.38_B_
	Rainy	192.69±5.02_A_	190.09±3.58_A_	197.51±5.58_A_
	Winter	152.74±3.63_C_	152.29±2.55_C_	162.14±5.32_B_
Potassium (mmol/L)	Summer	5.78±0.36_b__A_	4.09±0.04_c__A_	6.44±0.15_a__A_
	Rainy	3.44±0.21_b__B_	3.51±0.14_b__B_	4.07±0.16_a__B_
	Winter	5.41±0.20_a__A_	3.54±0.17_b__B_	6.16±0.37_a__A_
Chloride (mmol/L)	Summer	99.68±1.21_a_	92.70±0.35_b__B_	94.06±0.94_a_
	Rainy	106.53±7.20	94.28±0.87_B_	97.19±0.83
	Winter	113.60±3.89_b_	131.39±6.82_a__A_	100.18±5.07_b_
Calcium (mg/dl)	Summer	9.86±0.38_a__A_	8.77±0.31_b__A_	9.05±0.19_ab__C_
	Rainy	8.39±0.54_b__B_	7.46±0.36_b__B_	11.36±0.51_a__B_
	Winter	7.27±0.25_b__B_	6.97±0.28_b__B_	14.31±0.36_a__A_
Phosphorus (mg/dl)	Summer	4.85±0.19_B_	4.58±0.13_B_	4.85±0.35_A_
	Rainy	3.89±0.19_b__C_	5.02±0.15_a__B_	4.08±0.17_b_
	Winter	9.83±0.41_a__A_	7.20±0.53_b__A_	3.23±0.16_c__B_
Magnesium (mEq/L)	Summer	2.98±0.21_A_	3.18±0.10	2.45±0.12_a_
	Rainy	1.89±0.02_b__B_	3.05±0.29_a_	2.55±0.15_a_
	Winter	1.87±0.03_B_	2.98±0.23	1.97±0.02_b_

a-c Values in the same row with different superscripts differ significantly (p<0.05),

A-C Values in the same column with different subscripts differ significantly (p<0.05)

### Erythrocyte profile

Erythrocyte profile of pre-weaning Zovawk piglets was not influenced by season. The grower pigs were found to have significantly lower Hb, packed cell volume (PCV), mean corpuscular volume (MCV), mean corpuscular Hb (MCH) in summer and rainy season than in winter (p<0.05). The MCH concentration (MCHC) of grower pigs was lower in summer than in winter (p<0.05). The adult Zovawk pigs had higher TEC and lowered Hb during the rainy season as compared to that in summer and winter (p<0.05). Adult pigs had lower PCV and MCV in summer than in winter (p<0.05). During the rainy season, the adult pigs had PCV like in summer and winter however MCV was significantly lower than that in winter (p<0.05). MCH of adult pigs was significantly lower in the rainy season than in winter (p<0.05).

### Enzyme profile

During the pre-weaning period, SGOT, SGPT and ALP activities were not influenced by season. The SGOT activity of grower and adult pigs was higher in summer and rainy seasons than in winter (p<0.05). SGPT was influenced by season only during grower stage (p<0.05) with higher activity in summer as compared to that in the rainy season. In growers, ALP activity was found to be lower in winter than in rainy season (p<0.05). ALP activity of adult pigs was lower in summer than in rainy and winter seasons (p<0.05).

### Electrolyte profile

Zovawk pigs had higher Na and lower K during the rainy season (p<0.05). The pre-weaning and grower group had higher Na in summer than in winter. The grower pigs had higher K in summer than in winter (p<0.05). The Cl level was influenced by season only in grower group with higher values in winter as compared to summer and rainy seasons (p<0.05). The pre-weaning piglets and growers had higher plasma Ca during the summer than during the winter (p<0.05) while a reverse trend was recorded for Pi. The adult Zovawk pigs had higher plasma Ca during rainy and winter seasons (p<0.05) while a reverse trend was observed for Pi. The seasonal influence on plasma Mg of Zovawk was observed during the pre-weaning and adult periods with higher values in summer (p<0.05) than in winter.

## Discussion

From the present finding it was evident that hematological parameters were least affected by seasonal variation during the pre-weaning period while that of growers was most affected. The record of lower Hb, PCV, MCV, MCH and MCHC in the blood in growers during the summer and rainy seasons indicated that all the erythrocyte pictures except TEC showed variation due to stress due to higher THI (Table-1). As the grower pigs had been shifted from the mothers’ milk and subjected to grower diet, in high demand of nutritious diet in order to complement the overall growth and development of the body, higher THI during summer and rainy season were more stressful than winter as reported in other indigenous and crossbred pigs [5]. Higher THI when the heat load is more, one of the main behavior change recorded in pigs is a reduction in voluntary feed intake to minimize the metabolic heat production [20,21]. Although there was higher demand of nutrients for growth, the animals were probably consuming less feed due to more heat load [20], which resulted to drop in Hb, PCV, MCV, MCH and MCHC during summer and rainy seasons in grower group.

In case of adult animals, the pigs had lower Hb during the rainy season as compared to summer and winter, which might be due to high THI. As these animals are well-adapted animals, there was a compensatory increase in TEC during the rainy season in adult leading to PCV comparable to that of summer and winter. However, PCV and MCV of adult pigs were lower in summer as compared to winter due to thermal stress.

In the present investigation, the SGOT activity of grower and adult pigs was found to be higher in summer and rainy seasons than in winter (p<0.05). The SGOT activity increased from pre-weaning to grower during summer and rainy seasons (p<0.05), but not during winter. From the present finding, it could be assumed that both summer and rainy seasons caused higher SGOT in grower pigs as compared to pre-weaning pigs like in other indigenous pig and crossbred pigs [5]. SGOT activity of adult pigs was higher than that of pre-weaning pigs during the summer but not during the winter, which also indicated that in presence of higher THI, SGOT activity of Zovawk pigs also increased like in other pigs [5].

The presence of higher SGPT during summer in case of grower group might be due to stress as SGPT activity of grower pigs had been shown to increase in stress [22]. The finding of no seasonal influence on SGPT activity in case of adult pigs resembled the previous reports in Gunghroo, Niang Megha and Crossbreds [5]. The adult Zovawk pigs had higher SGPT activity than the pre-weaning and grower pigs as SGPT activity increases with increase in body mass [23,24].

ALP activity declined gradually with age in all the seasons as reflection of the decline in the demand of the enzyme for skeletal growth with age [25]. ALP activity of adult pigs was lower in summer than in rainy and winter seasons, which might be due to summer stress as ALP had been reported to decrease its activity when exposed to heat stress [5]. It had also been reported that in order to maintain stable blood calcium level with lowering serum levels of 25-hydroxyvitamin D during winter, ALP activity in elderly people increased during winter [26]. It could be possible that there was fall of ALP of adult Zovawk pigs due to heat stress during summer and increase in activity of ALP during winter in order to compensate the requirement of vitamin D.

The SGOT and SGPT activities of Zovawk pigs were in the ranges reported in Burmese pigs [27], Ghungroo and Niang Megha and crossbred pigs [5] but were lower than the activities reported in other exotic pigs [15]. ALP activity of Zovawk pigs resembled that of wild boar [11] but were lower than other crossbred pigs [28,29].

Minerals serve many important functions in pig nutrition. These range from structural functions in the bone to a wide variety of chemical reactions essential for maintenance, growth reproduction, and lactation. The presence of higher Na and lower K during the rainy season in all the age groups indicated an osmotic imbalance in these pigs during rainy season due to high humidity in addition to high ambient temperature. Activation of hypothalamo-pituitary adrenal axis by prevailing stress due to high temperature and humidity might have led to secretion of more aldosterone leading to retention of Na and excretion of K during the rainy season. Summer stress caused a higher concentration of Na in the pre-weaning and grower pigs and higher plasma K in growers. The concentration of plasma Na and K during summer and rainy season in growing period was indicative of dehydration with hypernatremia and hyperkalemia, which also caused lower PCV and MCV due to a decrease in intracellular fluid volume [30].

In this study, an increase in plasma Na and K during summer were accompanied by a decline in plasma Cl. The Zovawk pigs were found to have lower Cl in plasma during summer and rainy seasons than during winter with a significant difference recorded in grower pigs. Seasonal variation in plasma Cl could be due to seasonal variation in Cl excretion and parathyroid gland activity [31,32]. There is a close relationship between parathyroid hormone synthesis with 1,25-dihydroxyvitamin D in Ca homeostasis. Apart from the role of the parathyroid gland in homeothermy of Ca, parathyroid gland overstimulation also resulted to clinical hyperchloremia [33]. Rise in Cl level of grower pigs during winter season could be due to activation of parathyroid gland for PTH synthesis and secretion in order to compensate low synthesis of vitamin D during winter and restore low plasma Ca of the pigs during winter to normal.

The grower Zovawk pigs were at the fast growing stage, which required fine control in the balance between the thyroid and parathyroid glands in regulating endogenous calciferol and parathyroid hormone in exhibiting seasonal variation in plasma Ca and Cl. It had been shown that the pigs required vitamin D even when receiving diets with higher in calcium and phosphorus than were ordinarily recommended [34]. Exposure of pigs to sufficient solar radiation during December and January had been found to cure rickets [34]. Less exposure of the pre-weaning piglets and growers to solar radiation during the winter might have led to low vitamin D synthesis which might have resulted in drop in the plasma Ca in those pigs during winter and the combined effect of both might have led to activation of parathyroid gland during winter.

The Mg homeostasis is a result of balance between intestinal absorption and renal excretion with additional regulation by the adrenals, thyroids and parathyroid glands [25]. Mg ion had an effect on parathyroid secretory rate similar to that of Ca, but its effect was not equipotent to that of Ca [25]. The finding of higher plasma Mg during summer in the presence of higher Ca in the present study agreed the effects of Ca in control of PTH secretion, together with its preponderance over Mg in extracellular fluid suggesting a secondary role for Mg in parathyroid control [25].

## Conclusion

The erythrocyte profile, plasma enzyme activity and electrolyte status of Zovawk pigs were influenced by season. This study revealed a close association between the hemato-biochemical parameters in different seasons. The hemato-biochemical profile of grower pigs was most affected by seasonal variation, followed by adults and pre-weaning piglets. The changes in hemato-biochemical profile indicated that both summer and rainy seasons were stressful to Zovawk pigs. The estimated hematological and biochemical parameters could be used as measure of stress in Zavawk pigs.

## Authors’ Contributions

PM performed the analysis of blood samples and prepared the manuscript. TCT helped during the blood sample collection and the statistical analysis of the data. Both authors drafted and revised the manuscript. Both authors read and approved the final manuscript.
